# Correction: Antimalarial drug resistance molecular markers of *Plasmodium falciparum* isolates from Sudan during 2015–2017

**DOI:** 10.1371/journal.pone.0245336

**Published:** 2021-01-06

**Authors:** Maazza Hussien, Muzamil Mahdi Abdel Hamid, Elamin Abdelkarim Elamin, Abdalla O. Hassan, Arwa H. Elaagip, Abosufyan Hamattallah A. Salama, Mohammed H. Abdelraheem, Abdelrahim O. Mohamed

The sixth author's name is spelled incorrectly. The correct name is: Abosufyan Hamattallah A. Salama.

There is an error in affiliation 3 for author Abosufyan Hamattallah A. Salama. The correct affiliation 2 is: Faculty of Medical Laboratory Sciences, Kordofan University, Alobied, Sudan.

The word “marker” is misspelled in the article title, as well as in the Table 2 and Table 3. In all of these cases, “maker” should be “marker.” The correct title is: Antimalarial drug resistance molecular markers of *Plasmodium falciparum* isolates from Sudan during 2015–2017. The correct citation is: Hussien M, Abdel Hamid MM, Elamin EA, Hassan AO, Elaagip AH, Salama AHA, et al. (2020) Antimalarial drug resistance molecular markers of *Plasmodium falciparum* isolates from Sudan during 2015–2017. PLoS ONE 15(8): e0235401. https://doi.org/10.1371/journal.pone.0235401

In Table 2, the heading for column 1 should read: Drug resistance marker. In Table 3, the heading for column 1 should read: Drug resistance marker. Please see the correct Table 2 and Table 3 here.

**Table 2:** Frequency of mutant alleles in *Pfcrt* and *Pfmdr1*.

Drug resistance marker	Mutant alleles	N (%)
*Pfcrt* (n = 213)	M74I	153 (71.8%)
	N75E	153 (71.8%)
	K76T	153 (71.8%)
	A220S	154 (72.3%)
	Q271E	155 (73%)
	R371I	155 (73%)
*Pfmdr1* (n = 194)	N86Y	104 (53.6%)
	Y184F	171 (88.1%)
	D1246Y	3 (1.5%)

*Pfcrt*: *P*. *falciparum chloroquine resistance transporter* gene; *Pfmdr1*: *P*. *falciparum* multidrug resistance protein 1 gene.

**Table 3:** Frequency of *Pfcrt* and *Pfmdr1* haplotypes.

Drug resistance marker	number of mutations	mutation haplotype	KH n = 149	NH n = 29	G n = 21	NK n = 14	Total n (%)	P. value
*Pfcrt* (n = 213)	None	Wild Type CVMNK	48 (32.2%)	7 (24.1%)	5 (23.8%)	0 (0%)	60 (28.2%)	0.17
	Triple	CVIET	101 (67.8%)	22 (75.9%)	16 (76.2%)	14 (100%)	153 (71.8%)	0.17
*Pfmdr1 (*n = 194)	None	Wild Type NYD	9 (6.5%)	0 (0%)	4 (19.0%)	0 (0%)	13 (6.7%)	
	Single	**Y**YD	6 (4.3%)	1 (3.8%)	1 (4.7%)	1 (12.5%)	9 (3.1%)	
		N**F**D	51 (36.7%)	14 (53.8%)	9 (42.9%)	3 (37.5%)	77 (39.7%)	0.42
	Double	**YF**D	70 (50.4%)	11 (42.3%)	7 (33.3%)	4 (50.0%)	92 (47.4%)	0.48
		**Y**Y**Y**	1 (0.7%)	0 (0%)	0 (0%)	0 (0%)	1 (0.5%)	
	Triple	**YFY**	2 (1.4%)	0 (0%)	0 (0%)	0 (0%)	2 (1%)	

*Pfcrt*: *P*. *falciparum chloroquine resistance transporter* gene; *Pfmdr1*: *P*. *falciparum* multidrug resistance protein 1 gene; KH: Khartoum; NH: New Halfa; G: Gezira; NK: North Kordofan.
