# Supplementary material for: Antimalarial drug resistance molecular makers of Plasmodium falciparum isolates from Sudan during 2015–2017
Source: PLoS One. 2020 Aug 20;15(8):e0235401. doi: 10.1371/journal.pone.0235401 (PMC7446868; doi:10.1371/journal.pone.0235401)
Supplement: S3 File — (PDF) [file pone.0235401.s003.pdf]

Institutional Review Board

Date: 04/09/ 2016 IRB Serial **No: NU-IRB-16-09-04-1**

Ethical Approval Letter

This to certify that the research proposal entitled:

Project title:

Molecular Characterization of Antimalarial Drug Resistance Related Genes In *Plasmodium Falciparum* Isolated From Sudan.

Prepared and submitted by:

Maazza Hussien Mohamed Abdelrahman

Has been approved by the central Institutional Review Board, Al Neelain University.

Dr. Ameera Adam Mohamed

Institutional Review Board Member

بسم الله الرحمن الرحيم

University of Khartoum

Institute of Endemic Diseases

Research Ethics Committee

<u>Ethical clearance notification</u>

Date: 1st September, 2014

Applicants Name(s): Muzamil Mahdi Abdel Hamid

Department: PARASITOLOGY

**Reference No:**	**2/2014**
**Project Title:**	**Plasmodium falciparum/vivax malaria in central and eastern Sudan: Molecular epidemiology, burden, and development of Arcview GIS maps.**
**Principles investigator (s):**	**Dr MUZAMIL MAHDI ABDEL HAMID**
**Department (s):**	**PARASITOLOGY**

Thank you for submitting your application which was considered at the Ethics

Committee meeting number…Eight… on date…1st Sept. 2014………….

The following documents were reviewed:

a. Ethical Application form (dated)--------√--------------
b. Participant Information Sheet (dated)------ √----------
c. Consent Form (dated)---------------- √------------------
d. Approval scientific committee (dated) ------√--------
e. External Permissions (dated) --------√-----------------
f. Questionnaires (dated) -------√ ------------------
g. Others
h. specify:..

The Institute of Endemic Diseases- University of Khartoum and .Research Ethics Committee approve this study from an ethical point of view.

- Approval is given for three year
- Projects, which have not commenced within two years of original approval, must be re-submitted to the ethics committee.
- You must submit the annual progress report to the ethics committee
- You must inform your department when the research has been completed.
- If you are unable to complete your research within three years validation period , you will be required to write to the director of the institute and Ethics Committee (where approval was given) to request an extension or your will need tore- apply.
- Any serious adverse events or significant change which occurs in connection with this study and /or which may alter its ethical consideration must be reported immediately to Ethics Committee, and an Ethical Amendment Form submitted where appropriate.

Approval is given on the understanding that the "Guidelines for Ethical Research Practice" are adhered to.

Yours sincerely

DR. Faiza Mohammed Osman
Research Ethics Committee

Research Ethics Committee
Institute Of Endemic Diseases
University of Khartoum

Khartoum – Sudan. P.O.Box 102/TEL: +249-83-793265/ Fax:+249-83-779712
Email: erc@bioerc .org
