# Supplementary material for: Antimalarial drug resistance molecular makers of Plasmodium falciparum isolates from Sudan during 2015–2017
Source: PLoS One. 2020 Aug 20;15(8):e0235401. doi: 10.1371/journal.pone.0235401 (PMC7446868; doi:10.1371/journal.pone.0235401)
Supplement: S1 Data — (DOCX) [file pone.0235401.s004.docx]

**Supplementary data**

Sequencing data of *Pfcrt, Pfmdr1, Pfdhfr, Pfdhps, Pfk13, exonuclease* molecular markers of resistance are provided as supplementary data (Suppl.1).Sequences were deposited in the public repository European Nucleotide Archive (ENA) with accession numbers provided as supplementary data (Suppl.2).
