# Supplementary material for: Antimalarial drug resistance molecular makers of Plasmodium falciparum isolates from Sudan during 2015–2017
Source: PLoS One. 2020 Aug 20;15(8):e0235401. doi: 10.1371/journal.pone.0235401 (PMC7446868; doi:10.1371/journal.pone.0235401)
Supplement: Attachment — Submitted filename: Response to the reviewers Hussien et al 2020.rtf [file pone.0235401.s005.rtf]

Response to the reviewers: 

Reviewer #1:
Title of manuscript: Antimalarial drug resistance markers of Plasmodium falciparum isolates from Sudan during 2015-2017

Hussien et al.


General comments:
This manuscript covers an important topic with high relevance in public health. Since the current malaria control/elimination strategies rely to a greater extent on the use of antimalarials, it is important to monitor the performance of these drugs using different methods. The manuscript is well written but needs major improvements before it can be accepted for publication.

Specific comments:
1. Abstract:
a. The background is rather fragmented with two issues which are not well connected. Authors should revise and re-write the first statement into two separate sentences focusing on the significance and impacts of drug resistance on malaria control/elimination; and the use of molecular markers in the surveillance of drug resistance
# The background has been rewritten and revised as requested. 

b. Of the drug resistance genes mentioned on lines 61-63, I would suggest to include the type of drug(s) which are normally affected by mutations in each of the genes.
# The suggestion is will noted included in the conclusion of abstract mentioned in introduction part

c. How was multiplicity of infection determined and why? Since this is not reported in the results, authors may omit it in the abstract.
#   We have omitted the multiplicity of infection from the abstract. The details of the procedure were included in the methods section and the multiplicity of infection was reported in the results section of the manuscript.

d. The presentation of results with numbers and percentage is a bit confusing. Since authors reported frequency/prevalence of mutant alleles, I would suggest to give the percentage and  keep the number of samples with such alleles in brackets. I would also recommend to either use frequency or prevalence of alleles and not both.
#Done. We kept the percentage only and the detailed number of samples for each allele is given in the results section. We used the term frequency instead of prevalence throughout the text.

e. Conclusion: author report that no evidence of resistance in piperaquine, mefloquine and halofantrine were detected. They should explain the markers used to assess resistance to piperaquine. Since no prior information is given about the levels of drug resistance to chloroquine (CQ) and SP, it is difficult to understand why they think that CQ resistance has persisted and SP resistance has increased. No background information or levels of resistance to these drugs were given.
# The conclusion was rewritten to focus on outcome of resistance molecular makers. 

2. Background:
a. In the first paragraph, authors should provide more information on the epidemiological profile of malaria in Sudan, including the recent status as well as changes in malaria transmission (if any). They should also provide a brief account and history malaria treatment guidelines in Sudan. Such information will help the reader to understand the type of drugs which have been used in the country in the past 1-5 decades and the level of drug pressure exerted on parasite populations.

#The paragraph in introduction was rewritten to include information of prevalence of malaria  in Sudan and the study areas. The history of malaria treatment guidelines was included.

b. The entire section needs to be re-organized with each paragraph clearly addressing the same theme.
# Done as suggested.

3. Methods:

a. Why these study sites were selected for the study? Was this part of a different study or it was an independent study?
# The study sites were selected as those sites are malaria endemic and the epidemiology and entomology of malaria varies. Reference was included.
 Samples from Khartoum were collected specifically for this study while the samples from the other areas were archived dried blood spots from a previous study at the Institute of Endemic Diseases. The details were included in method section in manuscript

b. What research questions were being addressed in this study and what was the study design?

#The research question was to determine the frequency of malaria drug resistance molecular of P. falciparum at different sites. The study was descriptive cross sectional study. 

c. What were the inclusion and exclusion criteria for patients to be included in the study?

# All subjects with fever or other symptoms of malaria with a positive blood smear for P. falciparum asexual parasites. Exclusion criteria were severe illness and/or consent refusal.  

d. How were study subjects selected and sampled?

# All febrile patients attending an outpatient clinic with blood smear positive for P falciparum malaria  and given consent to participate was  included in the study during the period of the study. 

e. How was sample collection done?

#Blood samples were collected as fingerprick samples (50 ul) on Whatman 3 filter paper (Whatman International Ltd Maidstone, England) using aseptic conditions. The spots were allowed to dry, kept in a sealed plastic bag and stored at room temperature until used. Clarified in methods section.


f. How were the samples processed and stored in the field and lab before DNA extraction?

#Samples of blood drops on Whatman filter paper (50 ul) were allowed to dry at room temperature and then stored in plastic bag. 

g. What was the sample size, overall and in each of the four sites?

# Sample size= 226 (Khartoum  158, New Halfa 30, Gezira 23, North Kordofan 15). Provided in (Table 1)

h. Please provide the exact duration of sample collection (with start and end dates in months) 
 
# October 2015 till November 2017. Provided in methods section.

i. I would suggest to provide a rationale for selecting the genes which were assessed.

# These are the published genes used for the assessment of the drug resistance of P. falciparum malaria and were included in the Sanger Institute protocol. 

j. How was data analysis done?

# Data was entered in SPSS and frequencies of different genes and alleles were calculated. A paragraph of subtitle of data analysis was provided.


4. Results:
a. Please provide a baseline table showing the characteristics and the numbers of patients sampled in each of the four sites
# Table 1 was provided which include numbers of patients sampled in each of the four sites, male percentage, median age in years, multiclonal infections and MOI

b. What was the mean number of parasite clones (MoI/CoI) per site and overall?

# MOI was provided for each site and overall in (Table 1) and text.

c. Since 149 samples were from one site (Khartoum), I would suggest to group together all samples from all the remaining sites (table 2). Similar stratification should be done for the data presented in table 4. This should allow a meaningful comparison between Khartoum and other sites.
# This notion is added in methods section (study design and areas) any change will affect the primary objectives of the study.   
d. I would suggest to give a key under each table to describe all the abbreviations and symbols used.
# Abbreviations were added as suggested

e. The subheadings on exonuclease E415, k-13 and PBG can be combined into Pfk13 and other genes
# Well noted. Corrections were done as suggested.

5. Discussion and conclusion:

a. Authors mentioned that the results show no signs of reduction in CQ resistance in Sudan. It will be important to give a reason and explain why CQ resistance has persisted in Sudan.
# Yes, there is no sign in reduction of CQ resistance in Sudan. Officially, CQ has been stopped and replaced by AS/SP since 2005 and it's not legal to sell CQ. However persistence of resistance may indicate that CQ pressure is still on. We are added sentence in discussion to indicate this fact as we have no evidence for persistent use of CQ.

b. Why is SP resistance increasing in Sudan?
# We have added couple of sentences explaining significant of this mutation and the relevance of continued use of SP until 2017.

c. This study covered for sites but only few samples were collected in the other sites except Khartoum. Authors need to give reasons for this and provide an account of the limitations of this study.

# Explanation provided in the methods section, the Khartoum samples were collected specifically for this study while samples from other sites were archived samples.

d. If possible, authors should minimize repetition of results in the discussion.
6. Others comments.
a. In the key words, "molecular maker" should be changed to "molecular markers"
#Done as advised.

b. Authors should revise language and take care of typos
# Apologize for typos we have tried our best to clean them up

Reviewer #2: 
Abstract

1) State the method used in establishing multiplicity of infection
# Multiplicity of infection was omitted from abstract based on reviwer1 suggestion but included in the methods and results section of the manuscript.

2) Sentence, .....from four areas in Sudan from 2015-2017. This sentence is incomplete and also indicate the four areas and how they were selected
# Sentence was re-written and completed.

3) Highlight the most prevalent K13 mutations
# The most prevalent K13 mutations was highlighted (E433D)

4) Provide results for background mutations
#Parasite genetic background mutations (PGB) were included.

5) Conclusion needs to be revised to reflect the main outcomes of the study
# The conclusion was revised to focus on the outcome of resistance molecular makers analysis.

6) Summary of the results on mefloquine, piperaquine and halofantrine resistance are not listed in the abstract and thus should be omitted in the conclusion
# Summary of the results mefloquine and halofantrine resistance was omitted. Result of piperaquine resistance marker was included.

7) What were the resistance markers of halofantrine, mefloquine and piperaquine resistance
#  The abstract conclusion was revised to include the resistance markers studied. 

Introduction

8) line 109, sentence; ...the subsequence years,..... This should be adjusted to ......the subsequent years,.....
#Revised as suggested.

9) line 111, sentence;....providing bases for ......., This sentence should be revised to .....providing a basis for....
# Sentence was replaced as appropriate

10) Check the grammar of sentence, line; 118, 119, 122; in addition, this sentences are not clear
# We have rephrased the sentences and grammar was corrected

11) ACTs introduced in 2004 but treatment protocol change occurring in 2017, seems contradictory, please clarify
# ACTs introduced in 2004 as AS/SP which was changed in 2017 to AL this was better explained in manuscript

12) Cross check on the usage of the term 'artemisinin resistance', this should be adjusted to 'delayed parasite clearance' instead of artemisinin resistance
# Sentence was corrected as required and observed in all other places.

13) Line 135, 136 provide the reference
# Reference was provided.

14) Line 140-141; is not in line with the title of the manuscript
# We have adjusted the sentence to be in line with title of manuscript

Materials and Methods

15) How was Plasmodium falciparum confirmed in the study
  # Initially by Microscopic examination of Giemsa stained blood smears according to standard protocol. There were confirmed by amplification and sequencing of conserved regions of the Plasmodium parasite mitochondrial genome. Clarified in method section. 

16) How were the DBS cards prepared, dried, stored. How long was the storage period and how was quality ensured. A study by Schwartrz et al., 2015 showed that storage affect the quality of DNA from DBS
#DBS were from finger prick was spotted Whatman 3 filter paper (standard paper for saving dried blood samples for DNA extraction) and were stored at room temperature. The samples from Khartoum were stored for one year while the samples from other sites were stored for 3 years before shipping to Sanger centre in UK. The quality checked was ensured by Sanger Institute before proceeding to species confirmation and amplicon sequencing.

17) Describe the study setting, health facilities where the samples were collected from
#The samples were collected from primary health care centres at respective sites

18) How was blood collected (quantity)
 # 50 ul. Described in the method section.
19) Line 158, define the study participants (sex, age etc)
  #The age and sex were described in the method section.

20) line 159, describe how the Giemsa stain was diluted and how the test was done
# 10% Giemsa stain the protocol for slide preparation and staining was referenced

21) Line 161-162, provide ethical clearance number and attach the ethical clearance form
 #The ethical clearances details and numbers were provided and attached.

22) Provide details of how parasite DNA was extracted and how quality was ensured during the extraction process
# All DNA extraction, quality verification and sequencing were performed by Sanger institute.

23) Provide listing of all the primers and the adapter sequences used in this study. This was not provided and so the accuracy of the genotyping could not be cross checked which is a major limitation of this work

#Amplicon sequencing procedure including the primers and adapters for sequencing will be published separately Wellcome Sanger Institute, UK.  However, we provided the maximum possible information allowed for us to include in this manuscript and we have indicated that the source of information was a manuscript under in preparation by Sanger Institute.  Two references describing part of the procedure are provided in the methods section

24) Describe the source of the primers used (were the primers from previous studies or you generated them).
#The protocol of sequencing and the primers were generated by Sanger Institute and will appear in the upcoming manuscript. 

25) Data management, Data analysis, are missing and should be included
  #Data management and data analysis was added in the methods section.

26) If the primers were generated please describe how this was performed
  # As above.

27) How was the sample size of 226 determined, please justify this sample size. Does this sample size give the study the necessary power to measure the intended outcome?
# The sampling was convenient sampling for Khartoum site and archived dried blood samples from the other sites. The frequency of different drug resistance markers and definitely meaningful for this sample size.

28) How quality control was ensured in the laboratory tests is not described making it difficult to believe in the findings. Please describe how quality was ensured throughout the laboratory processes
# We provided supplementary data for the amplicon results of all samples included which we received from Snager Institute. All amplicon sequencing data was deposited in European Nucleotide Archive (ENA), which fulfills the request for deposition in a public repository; This was mentioned in the manuscript and ENA accession numbers are provided as supplementary file.
Furthermore if you have question on integrity of the finding you can contact MalariaGEN group at Wellcome Sanger Institute ( Dr     Sonia Gonvalves, Email:   sg19@sanger.ac.uk).

Results

29) For all the proportions/prevalence values, please provide the fractions (numerator and denominator)
# Numerator and denominator were provided throughout the results.
30) Line 201, which mutations are being referred to ....
# Mutation is Pfcrt indicated in the subtitle and first line

31) The number of successful amplifications for K13 mutations is too low, please explain the high observed failure rate
#It is unfortunate that k13 amplifications had high failure rate. However, we assured this is not due to the quality of DNA which gave excellent results for other markers. We will wait until the protocol is published by MalariaGen group at Sanger and we will discuss with them this rather low yield.
32) What was the most prevalent K13 mutation

# E433D was the most prevalent K13 mutation. Revised in the text.


Discussion

33) line 258....chloroquine as reported earlier, what does this mean?
# As reported earlier that sensitive strain of parasite will dominate. Corrected in text.

34) Please clarify on what you mean on line 262-263
# clarified by splitting it in to two sentences.

35) There was no intentional screening for markers of resistance to mefloquine, halofantrine and piperaquine and thus this should be removed from the discussion
# In this amplicon screening analysis this markers are included and the result were obtained indicating absence of resistance to these drugs, of them only piperaquine is of concerned to us as its partner  to DHPPQ the second line of treatment in Sudan.

36) Line 277-284, please explain the result that was found
# We have added couple of sentences explaining significant of this mutation and the relevance of continued use of SP until 2017

37) Line 295-296, has any study been done on this before??
# Reference has been added for previous in vitro studies to evaluated novel mutation

38) The result on background mutations need to be explained

# Explanation provided for the sentence concerning genetic background

Conclusion

39) This should be restricted to genotyping resutls not phenotypic as phenotypic resistance was not measured
# The conclusion was rewritten focusing on genotyping results as suggested.

40) What do the authors mean in the sentence, .....good point that this mutation has not been recorded in Sudan....?, Please clarify
# The sentence was rephrased  to explain the importance of the absence of exonuclease mutation.

Acknowledgement

41) The fact that the DBS were all provided by the department was not mentioned in the methods section, which is a major omission. Please provide details as pointed out in my comments in the methods section. On the ethical point of view what was the purpose of collection of this samples by the department, were they for research and if so please provide ethical clearance of the primary study. Also indicate if the primary study participants consented for their samples to be used in further studies.

# Acknowledgement was re-written and the necessary information was included to methods section. Samples from Khartoum were collected specifically for this study while the samples from the other sites were archived dried blood spots from a previous study at the Institute of Endemic Diseases. The institute always consent participants of different studies on the use for their archival blood spots to be used for further studies. Detailed information on DBS samples provided by Institute of Endemic Diseases including ethical issues is stated in the methods section.

References

42) The references are adequate, provide the missing references as indicated in earlier sections of my review
# The missing references were provided.


Reviewer #3: 
This is highly validated research for cross-sectional drug resistance markers study in Sudan. Even this ms was not described in vitro of relevant samples and clinical drug sensitivity of patients, it is highly informative for malaria research fields, especially prevalence of drug resistance in one of East African countries.

Major comments>
1. Authors should described the relavant drug resistance and used standard resime drug in clinics of field sites in Sudan for P. falciparum and explained relationship of SNP and used drug currently.
# We have mentioned the sequence of malaria treatment protocols changes in Sudan due to resistance from Chloroquine to AS/SP and AL. We have added in the discussion the suitability of the present treatment AL as first line and DHA-PPQ as a second line as there is no resistance to any of the combination members.

2. What about prevalence of malaria in study areas? Pf only or mixed cases with non-Pf malaria species for treatment. If patients treated and exposured with CQ for Pv previously, how to explain about drug sensitivity?
# We have added this information in the introduction part and references were added. 
In the national protocol chloroquine was replaced since 2005, and cannot be obtained officially from pharmacist. However, the persistence of resistance is worth noting. 

3. What about in vitro drug sensitivity and/or drug sensitivity clinically for field isolates and relationship with SNP about each drug marker? Please describe in manuscript.
This study is not an efficacy study and we did not perform any in vitro testing so we cannot associate the SNP markers with any treatment outcome.

Minor comments>
1. Please correct some of italic font text and parasite name.
#   All required are observed. 

2. Were there any low drug sensitivity samples from field isolates?
#  This is not in vivo efficacy study. 

3. Were there any places or patients in local clinics or personal TX still use CQ for Pf instead of ACT Tx?
# None, we know of. 

Dr Muzamil Mahdi Abdel Hamid
MSc, PhD     Associate Professor
Professor Abdelrahim Osman
MBBS, PhD
