# Supplementary material for: Antimalarial drug resistance molecular makers of Plasmodium falciparum isolates from Sudan during 2015–2017
Source: PLoS One. 2020 Aug 20;15(8):e0235401. doi: 10.1371/journal.pone.0235401 (PMC7446868; doi:10.1371/journal.pone.0235401)
Supplement: Attachment — Submitted filename: COMMENTS ON THE REVIEW OF PONE-D-19-3468-R1.doc [file pone.0235401.s006.doc]

**COMMENTS ON THE REVIEW OF PONE-D-19-3468_R1**

Thanks editor for the opportunity given to me to review the above manuscript. From the initial review it is clear that the authors did make significant adjustments however, there are still areas that need to be addressed to make the manuscript suitable for publication. Here below are the highlights of the areas that need to be addressed.

1. The sampling procedure used during field data collection should be described. How individual patients were selected for inclusion into the study
2. Describe the quality control done by the Sanger Institute Welcome Trust
3. Describe the procedure of Nested PCR and illumina Miseq used
4. Comprehensive grammatical review is needed for this manuscript
5. Specify the conserved mitochondrial gene used in confirming *P. falciparum* parasite infection and provide details of how this was performed
6. What concentration of Giemsa stain was used in the laboratory diagnosis of malaria (line 178-179)
7. Line 193-914; the information provided in this manuscript must be stand alone. Therefore, the authors should provide the specifications/details of all the primers including the manufacturer. All the primers used in the PCR/Sequencing/Multiplex PCR and how they were designed for each of the SNPs assessed in this study
8. Provide reference for the Nested amplification protocol used in this study
9. Provide details of how the raw sequences were analyzed to establish the mutations/SNPs in this study
10. Line 228-229; identify/specify the mutations being referred to

**Thanks**

**Moses Ocan (PhD)**
