# Supplementary material for: Antimalarial drug resistance molecular makers of Plasmodium falciparum isolates from Sudan during 2015–2017
Source: PLoS One. 2020 Aug 20;15(8):e0235401. doi: 10.1371/journal.pone.0235401 (PMC7446868; doi:10.1371/journal.pone.0235401)
Supplement: Attachment — Submitted filename: Answers to the reviewers PLOSONE-D-19-3468-R1.docx [file pone.0235401.s007.docx]

**COMMENTS ON THE REVIEW OF PONE-D-19-3468_R1**

Thanks editor for the opportunity given to me to review the above manuscript. From the initial review it is clear that the authors did make significant adjustments however, there are still areas that need to be addressed to make the manuscript suitable for publication. Here below are the highlights of the areas that need to be addressed.

Response Reviewer #1:

1. In the background, I would recommend to replace reference #1 with the most recent WHO report of 2019

\# We have updated the malaria WHO report 2019 reference as advised and updated the prevalence of malaria in the manuscript.

2. In the results, lines 225-230, 244-247, and 253-256 present results of hyplotypes in Pfcrt, Pfdhfr and Pfhps, resppectively. Authors should change the presentation from genotypes to haplotypes to be specific.

\# We made the required changes as advised.

3. Tables 4 and 5 were interchanged. This need to be corrected

\# Corrected.

4. The manuscript still needs language corrections which authors might seek support from native English speakers.

We have done a comprehensive revision throughout the manuscript. Moreover, Sanger staff kindly have offered the final proofreading of the manuscript.

Response to Reviewer #2:

1. The sampling procedure used during field data collection should be described. How individual patients were selected for inclusion into the study

\# The sampling procedure was described. Inclusion criteria stated.

2. Describe the quality control done by the Sanger Institute Welcome Trust

\# In the methods we have described the DNA extraction procedure from DBS samples. Also we have described the QC for quality and purity of amplicons as well concentration before proceeding to library preparation.

All quality control steps will appear in full details in upcoming methodology manuscript under preparation by Sanger Institute, UK. For more information feel free to contact Dr Christopher and Dr Jacob [ecj9@sanger.ac.uk](mailto:ecj9@sanger.ac.uk) and Dr. Kimberly Johnson	[kim.johnson@bdi.ox.ac.uk](mailto:kim.johnson@bdi.ox.ac.uk)	from MalariaGen, Sanger Institute in the UK.

3. Describe the procedure of Nested PCR and illumina Miseq used

\# We have described the procedure of nested PCR and illunmna Miseg used in short as permitted by Sanger group in UK. We have given a reference for the selective whole genome amplification

(sWGA) procedure. All detailed steps of the procedure of nested PCR and and illumina Miseq will appear in the upcoming methodology paper by the MalariaGen Sanger group. We have provided it as permitted by Snager.

4. Comprehensive grammatical review is needed for this manuscript

\# We apologize for the grammatical mistakes and typos. We have done comprehensive revisions throughout the text (as track changes). Moreover, Sanger staff kindly have done the final revision and made final proofreading and corrected extensively the methods section.

5. Specify the conserved mitochondrial gene used in confirming *P. falciparum* parasite infection and provide details of how this was performed

\# *Plasmodium* species was determined by analyzing highly conserved segments of the parasite mitochondrial genome common to all 5 human infecting species (*P. falciparum, P. vivax, P. ovale, P. malariae,* and *P. knowlesi* . Reference was given.

6. What concentration of Giemsa stain was used in the laboratory diagnosis of malaria (line 178-179)

\# The concentration of Giemsa stain used was 10% (included in the methods)

7. Line 193-914; the information provided in this manuscript must be stand alone. Therefore, the authors should provide the specifications/details of all the primers including the manufacturer. All the primers used in the PCR/Sequencing/Multiplex PCR and how they were designed for each of the SNPs assessed in this study

\# The exact primers (PCR/Sequencing/Multiplex PCR) design, QC criteria and sequences will be published elsewhere (Jacob et al., manuscript in preparation). We apologize we cannot disclose it in this manuscript.

8. Provide reference for the Nested amplification protocol used in this study

\# The reference for using selective genome sequencing was provided, All aspects and details for the nested amplication protocol will be published as described above.

9. Provide details of how the raw sequences were analyzed to establish the mutations/SNPs in this study

\# All bioinformatics analysis were performed at Sanger. The sequencing analysis was decribed in the manuscript. In brief samples reads (190-250 bp) were de-plexed using the multiplexing adapters and individual CRAM files were aligned to a modified amplicon reference genome (*Pf*3D7). Genotyping was done using bcftools as well as custom scripts to filter and translate genotypes into drug resistance haplotypes and species determination.

10. Line 228-229; identify/specify the mutations being referred to

\# The mutations being referred to was the *Pfcrt* mutant haplotype (CV**IET).** It was corrected in the paper.

**Thanks**

**Moses Ocan (PhD)**

Thank you,,,

Dr Muzamil and Prof Abdelrahim

Parasitology Dept.

University of Khartoum

Sudan
