# Supplementary material for: Antimalarial drug resistance molecular makers of Plasmodium falciparum isolates from Sudan during 2015–2017
Source: PLoS One. 2020 Aug 20;15(8):e0235401. doi: 10.1371/journal.pone.0235401 (PMC7446868; doi:10.1371/journal.pone.0235401)
Supplement: Attachment — Submitted filename: Answers to the reviewers PONE-D-19-34681R2.docx [file pone.0235401.s008.docx]

**PONE-D-19-34681R2**

**Response Reviewer #1:**

Reviewer #1: 1. Authors' affiliations should be listed sequentially according the order they appear in the list of authors. This will help to reduce confusion caused by jumping numbers in the affiliations of authors.

\# Authors' affiliations should be listed sequentially according the order they appear in the list of authors.

2. The information presented on liness 104-107 cannot be prevalence but rather the proportion of infections with Pf, Pv or other species of malaria parasites. Sudan cannot have that high prevelance of parasite infections.

\# corrected to proportion of infections.

3. On line 180, the word "atteding out patient department" is repeated twice.

\# corrected so as avoid word repetition.
4. On line 301, the first statement is incomplete or hanging. I would suggest to merge it with the next statement.

\# The sentence was corrected and completed.

5. Authors should correct minor language problems and typos.

\# The minor language problems and typos were found and corrected and the manuscript was extensively revised.

**Response Reviewer #2:**

Reviewer #2: All comments raised in the previous review have been addressed by the authors. However, the authors could have provided the details of the primer sets used and their design

\# Thank you. As stated previously and confirmed by Sanger Institute, all details of primer sets and design will appear in the upcoming methodology paper by the MalariaGen Sanger group. This has been stated in the methods section.
